# Multi‐modal functional MRI to explore placental function over gestation

**DOI:** 10.1002/mrm.27447

**Published:** 2018-09-21

**Authors:** Jana Hutter, Paddy J. Slator, Laurence Jackson, Ana Dos Santos Gomes, Alison Ho, Lisa Story, Jonathan O’Muircheartaigh, Rui P. A. G. Teixeira, Lucy C. Chappell, Daniel C. Alexander, Mary A. Rutherford, Joseph V. Hajnal

**Affiliations:** ^1^ Centre for the Developing Brain King's College London United Kingdom; ^2^ Biomedical Engineering Department King's College London United Kingdom; ^3^ Centre for Medical Image Computing and Department of Computer Science University College London United Kingdom; ^4^ Women's Health Academic Centre King's College London London United Kingdom

**Keywords:** development, diffusion MRI, placenta, postprocessing, pregnancy complications, relaxometry

## Abstract

**Purpose:**

To investigate, visualize and quantify the physiology of the human placenta in several dimensions ‐ functional, temporal over gestation, and spatial over the whole organ.

**Methods:**

Bespoke MRI techniques, combining a rich diffusion protocol, anatomical data and T2* mapping together with a multi‐modal pipeline including motion correction and extracted quantitative features were developed and employed on pregnant women between 22 and 38 weeks gestational age including two pregnancies diagnosed with pre‐eclampsia.

**Results:**

A multi‐faceted assessment was demonstrated showing trends of increasing lacunarity, and decreasing T2* and diffusivity over gestation.

**Conclusions:**

The obtained multi‐modal acquisition and quantification shows promising opportunities for studying evolution, adaptation and compensation processes.

## 1 INTRODUCTION

The placenta has the crucial role to deliver oxygen and nutrients to the growing fetus. It furthermore removes waste products and fulfils important endocrine and immunity functions. The involvement of placental deficiencies in major pregnancy complications including miscarriage, pre‐eclampsia (PE) and fetal growth restriction (FGR) is well established.^1^, ^2^ PE is characterized by maternal hypertension and proteinuria and FGR by fetal growth below the genetic potential. These conditions are responsible for significant fetal and maternal morbidity and mortality.

Undiagnosed FGR is the most common risk factor for stillbirth.^3^ Pre‐eclampsia affects 2–8% of pregnancies–varying with geographic, social, economic, and racial factors and is associated with significantly increased prematurity as well as lifelong implications for the mother.^4^ Early detection is essential for efficient treatment including close monitoring and planned preterm delivery where indicated. However, clinical screening and diagnosis of placental disorders with uterine artery and umbilical cord ultrasound currently lacks specificity and sensitivity: The majority of FGR cases occur in low risk pregnancies and remain undetected until they present with signs of fetal distress or with unexpected stillbirth.^5^, ^6^ For PE, no single prediction test has demonstrated a sufficient predictive value^7^ to enter clinical use.

Understanding disease aetiology and developing sensitive methods for identifying these conditions early could contribute to improved management in the future. The complexity of the underlying disease suggests that an approach combining several techniques would be required—including metabolic, biochemical, immune, genetic and imaging studies. Imaging studies would ideally allow visualization along two dimensions:

**(A) Functional (Maternal to fetal)**. While the placenta is anatomically divided in 10‐40 units, separated by the septa and often referred to as cotyledons, a functional placental unit is defined by the presence of a spiral artery inlet. The oxygen and nutrient exchange from maternal to fetal side, occurs within these units. Oxygen‐rich maternal blood from the uterine arteries enters the lobule from the spiral arteries and perfuses the inter‐villous space, where the large surface provided by the branched fetal villi allows efficient diffusive transport across a thin membrane.

**(B) Spatial (over the whole organ)** The composition of the placenta with about 10–40 of these lobules with a significant degree of heterogeneity opens another important study dimension. The whole organ is relevant as compensation mechanisms and the fraction of functional exchange volume might influence its function.

For both these dimensions, studying the temporal progression over gestation is of key importance as the placenta changes dramatically throughout gestation to meet the growing needs of the fetus. The fetal villi undergo branching and angiogenesis to promote effective oxygen and nutrient exchange creating an ever denser network of fetal terminal villi around the maternal spiral arteries. In addition, fibrin deposition in the septa increases and the cell columns become deeply invaginated in the basal plate^8^ leading to an increasingly more apparent granularity or lobulation of the placenta.

Current clinical tools^9^, ^10^, ^11^ are not able to assess these dimensions in combination. The most promising US approaches are 3D‐power Doppler^12^, ^13^ and contrast‐enhanced US,^14^ but neither allows whole‐organ assessment in later gestation and are not able to depict the key units of exchange.

These are exciting opportunities for MRI to visualize and quantify the complexity of the underlying structural and functional processes and monitor their evolution over gestation, but this requires development and application of an imaging approach combining techniques sensitive to several functional layers. Previous MRI studies^15^ have visualized placental sub‐functions: oxygenation,^16^, ^17^, ^18^, ^19^, ^20^, ^21^, ^22^ perfusion^23^, ^24^, ^25^, ^26^, ^27^ and apparent diffusion,^28^, ^29^, ^30^ which provide confidence that MRI can be effective. Contrast‐enhanced techniques include oxygen‐enhanced MRI^21^, ^31^, ^32^ and dynamic contrast enhanced (DCE) techniques.^24^ Thereby,^22^, ^24^, ^33^ are studies using non‐human primate models.

While some studies combine several functions, such as perfusion and oxygenation,^24^, ^33^ further work towards visualizing even more sub‐functions at the same time is required.

Quantitative features have been obtained previously using placental relaxometry,^16^, ^18^, ^19^, ^20^, ^22^, ^33^, ^34^ BOLD MRI,^35^ dynamic contrast‐enhanced MRI^24^, ^33^ or diffusion MRI.^28^, ^29^, ^30^ Anatomical imaging of the placenta has been performed by numerous groups and several attempts have been done to objectively assess placental appearance,^36^ but to our knowledge no signature was so far able to quantify changes across gestation nor attempted quantitative assessment.

This study presents **(I) a bespoke Multi‐modal MRI acquisition** ‐ targeted to assess the placenta along the two described dimensions ‐ functional and spatial throughout gestation ‐ within a clinically feasible MRI examination of ≤20 min without requiring administration of contrast agents. The proposed acquisition protocol is combined with **(II) a dedicated post‐processing pipeline** to exploit these different information dimensions and obtained complementary MRI contrasts. Finally, **(III) quantitative features** are explored which are able to capture the evolution of these findings over gestation while highlighting spatial heterogeneity.

## 2 METHODS

### 2.1 Bespoke Multi‐modal MRI acquisition

Anatomical imaging of the placenta is the first step to a thorough assessment and is required to plan the functional techniques. A T2‐weighted sequence was selected to be sensitive to placental maturation, including increasing lobularity and granularity, while being robust to maternal breathing and fetal motion. Therefore, a 2D TSE with an echo time of 200ms was employed with an in‐plane resolution of 1.5 mm × 1.5 mm, a slice thickness of 2.5 mm with an overlap of 0.5 mm. T2w sequences were acquired both in the coronal and sagital plane in relation to the maternal pose to allow for later 3D reconstruction with slice‐to‐volume (SVR) techniques^37^ and derived volume calculation. The FOV equals 300 × 360 × [100‐‐200] mm (FHxRLxAP, coronal) and 300 × 340 × 300 mm (FHxAPxRL, sagittal), TE = 190 ms, TR = 16 s, SENSE 2.5, partial Fourier 0.625.

For both functional scans, the scan orientation was chosen as coronal to the mother as this provides both the highest efficiency in terms of coverage for all possible placental locations and the easiest in‐plane orientation to assess the heterogeneity over the whole organ.

To visualize the key function of the placenta—the transfer of oxygen—a functional sequence sensitive to oxygen concentration is required. Based on previous studies,^16^, ^18^, ^19^, ^20^, ^22^, ^33^ T2* mapping was selected due to its sensitivity to concentration of de‐oxygenated hemoglobin. Multiple echo times in the same anatomical position are required for T2* fitting, therefore a multi‐slice, multi‐echo gradient echo sequence (MEGE) was selected, acquiring all required echo times closely together in time.^38^

High isotropic spatial resolution is required to avoid partial voluming and thus underestimating of oxygen concentration in the expected small area of high oxygen concentration close to the small scale of the artery inlets (0.5 mm^39^). The need to balance the need for high spatial resolution with the limitations imposed by an acceptable acoustic noise level for fetal use and the requirement for a short echo spacing to allow robust fitting were explored in preliminary experiments, leading to the final choice of an echo spacing of 0.98 ms, resolution of 2 mm isotropic (corresponding as well to the diffusion acquisition), SENSE 3.5, partial Fourier 0.65.

Preliminary experiments were performed on 6 volunteers spread over the selected range 20 to 40 weeks gestation to investigate the spread of T2* values. These revealed an expected range of T2* values between 10 up to 200 in the central area of the hyperintense regions with a strong negative correlation with gestational age in line with previously reported values (e.g.^18^). The read‐out limitations fixed the minimal achievable initial echo time to 13.8ms and the inter‐echo spacing to 56.6. To cover the expected T2* values five echos were used with resulting echo times of [13.81, 70.40, 126.99, 183.58 and 240.17] ms.

Microstructural properties of interest include the fetal villi branching, length and distribution within the IVS as well as the perfusion in the maternal basal plate. Advanced diffusion models, which can provide a means to investigate these properties, require data with high angular resolution sampled on a range of diffusion strengths and at high spatial resolution. Approaches such as Intra‐voxel incoherent motion (IVIM)^40^ provide a means to estimate perfusion and require the acquisition of multiple *b*‐values in the low‐b regime. Recent work has shown that anisotropy is present in both the perfusion (i.e low *b*‐value) and diffusion (high *b*‐value) placental dMRI signal components.^41^ We therefore designed a bespoke diffusion weighted single shot EPI sequence. This has 3 gradient directions at *b* = 5, 10, 25, 50, 100, 200, 400, 600, 1200 and 1600 s/mm^2^, 8 directions at *b* = 18 s/mm^2^, 7 directions at *b* = 36 s/mm^2^, and 15 directions at *b* = 800 s/mm^2^, in addition 6 volumes at *b* = 0 s/mm^2^ were acquired interspersed through the acquisition.

For similar acoustic noise considerations and to facilitate registration the same resolution of 2 mm isotropic was chosen, together with a TE = 95 ms, TR = 7 s, SENSE 2, partial Fourier 0.7.

Contrary to the T2* mapping sequence, the high number of volumes and thus long acquisition time led to a different FOV in the AP direction for the diffusion scan. The number of slices is chosen to cover the placental volume only, leading to a FOV of 300 × 360 × [80−200] mm and thus variable acquisition times between 6 and 12 min.

### 2.2 Postprocessing

**Figure 1:**
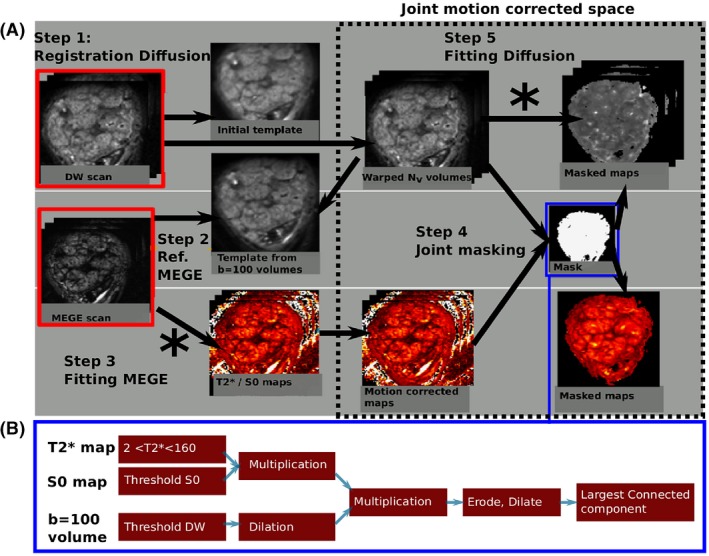
(A) The three steps of the post‐processing algorithm for functional data are illustrated. The stars signify the points in the pipeline where the acquired data is fitted to its respective model. (B) The masking step is depicted with the involved sub‐steps

Motion artefacts, originating both from fetal motion and maternal respiration, constitute a major challenge specifically for longer functional scans. The use of rapid slice selective methods allows individual slices to be acquired fast enough to freeze motion, but breathing motion introduces both displacements and non‐rigid deformation of the placental structure which needs to be addressed. To allow assessment of the whole cascade of events, the information of the different functional modalities needs to be represented in the same space. This space, however, does not correspond fully to the anatomical space, as both functional modalities are acquired with EPI based techniques leading to distortion effects. In addition, quantitative factors are extracted for all modalities. The whole process is described in the following and in Figure 1. One important difference between the processing of the MEGE and the diffusion data is the order in which steps are applied. The diffusion data is motion‐corrected first then model fitted, while the fits are performed first for the MEGE as the resulting parameter maps are used for the joint masking.

#### 2.2.1 Step 1—registration diffusion

Non‐rigid multi‐contrast registration is used to generate a template from the obtained 64 diffusion‐weighted volumes.^42^ This template represents the volume requiring the smallest deformations to transform all the individual volumes into this space. The obtained transformations are used to warp all diffusion volumes into this template space.

#### 2.2.2 Step 2—registration MEGE

In the next step, multi‐contrast registration is used to obtain the transformation which is required to map the MEGE volume to the created template from the dMRI data. For both MEGE and dMRI data, several co‐registered volumes are available: For MEGE, the available 5 echo times are all acquired close enough to freeze motion and thus considered intrinsically registered. Translations between subsequent echos were reported as minimal for fetal brain MEGE^38^ and are even smaller for the placenta, which does not experience autonomous movement but mostly slow respiratory displacements and displacements from uterine contractions. No correction for the contraction artefacts was included due to the unpredictable nature of these. All images were visually assessed for placental shape changes and patients with visually striking contractions were excluded.

The MEGE and dMRI volumes are then registered using the volumes with the closest structural similarity as source and target. For this registration step, the b = 100 dMRI volume and the second echo with an echo time around 60ms from the MEGE both depicting well the heterogeneous lobular appearance which act as natural landmarks.

#### 2.2.3 Step 3—fitting MEGE

Voxelwise T2* and proton density maps are calculated from the MEGE data by fitting a mono exponential decay model to the measured intensities (*S*_*i*_) and their respective echo times (*TE*_*i*_):

$$S_{i}\left(TE_{i}\right)=S_{0}e^{-\frac{TE_{i}}{T2}}$$

where *S*_*i*_ is the signal at echo *i* with *i*  ∈  [1,…,5] and *S*_0_ the signal at *TE* = 0. Ordinary non‐linear least squares regression was used for the fitting. The image data at the first echo time (*S*_1_) was used as the starting values for *S*_0_ and the T2* value was initialized with *T*2* = 100. To speed up the fitting procedure in voxels corresponding to noise, the allowed T2* values were restricted between [0, 400].

#### 2.2.4 Step 4—joint masking

Once all functional data is co‐registered, an automatic masking algorithm can combine information from both modalities and a‐priori knowledge about tissue differences. The MEGE data fitting is performed first, as the obtained T2* values are valuable in the masking step. All following steps were performed using MRTRIX3: First, a parameter‐free threshold method^43^ is employed on the proton density map. The T2* images are thresholded between [2 ms and 160 ms]. Subsequent multiplication of both insures that only voxels meeting both conditions are retained in *mask1*. The dMRI *b* = 100 volume is threshold^43^ followed by application of dilation leading to *mask2*. Subsequent multiplication of these two binary masks *mask1, mask2* retains only the voxels where both conditions are met and form the initial mask. This is refined by subsequent erosion, dilation and connected largest component steps. The entire process is illustrated in the Figure 1(B). The obtained mask is refined manually to separate placental volume and uterine wall regions‐of‐interest (ROI).

The T2* maps over the entire placental volume are calculated to quantify spatial inhomogeneity. Furthermore, a histogram analysis allows a more detailed depiction of whole organ T2* effects. The anatomical data is currently segmented manually.

#### 2.2.5 Step 5—fitting diffusion

The diffusion tensor, and hence apparent diffusion coefficient (ADC) and fractional anisotropy (FA) maps, are estimated from the dMRI data (all *b*‐values) with the MRtrix3 function dwi2tensor.^44^ We further analysed the data using zeppelin‐zeppelin: a two‐compartment microstructural model which allows for anisotropy in both fast‐ (associated with perfusion) and slow‐(primarily associated with diffusion) attenuating signal components.^44^

We fit the zeppelin‐zeppelin model using maximum likelihood estimation assuming Rician noise. This consists of a grid search, followed by nonlinear regression as described in.^45^ To increase the robustness of parameter estimates we randomly perturbed the initial values for nonlinear regression 20 times, and chose the parameter set which maximized the log‐likelihood.

Fitting this model voxelwise yields: *Dp* and *D*, two cylindrically symmetric tensors (i.e. zeppelins) representing perfusion and diffusion compartments respectively; and the perfusion fraction, *f*, which reflects the proportion of blood flowing in capillaries. ROIs for the placenta and basal plate were drawn manually. This was required as the previously obtained mask includes both regions, the diffusion properties, however, differ as they depict different processes.

#### 2.2.6 Structural quantification

The maturation of the placenta in terms of granularity was accessed on a mid‐parenchymal slice using a modified lacunarity index as presented in^46^ with the modifications to work with non‐binary grey‐scale images and the local mean within each box. The details of the method are in the Supporting Information.

### 2.3 Data acquisition and study population

Fifty‐seven pregnant women were recruited for a fetal MRI examination as part of ongoing fetal studies at St. Thomas’ Hospital. These included two women diagnosed with pre‐eclampsia according to clinical guidelines who were scanned at 33 and 34+1 weeks GA respectively. The remaining women were recruited from un‐complicated pregnancies showing—at the time of the scan—no signs of pre‐eclampsia. Informed consent was obtained and then all participants were imaged in a supine position^47^ on a clinical 3T‐Philips Archieva Scanner using a 32‐channel cardiac coil.

All datasets were processed, but only the anterior and fundal placentas were included into the quantitative measures to assure similar SNR patterns. See Supporting Information Figure S1 for an exemplary case of a lateral placenta, illustrating the SNR difference between anterior and posterior part. Furthermore, only datasets with at least one of the two functional modalities (T2^*^, DMRI) were selected. Finally, results from 24 MEGE, 24 anatomical and 16 DMRI datasets were obtained. Further 7 subjects had DMRI data, but not the full proposed diffusion scheme.

## 3 RESULTS

As stated above, all data sets were processed, but quantitative information was only obtained from the anterior placentas, because of the lower SNR in the posterior placentas. Example of (unprocessed) coronal and sagittal images from a lateral placenta are shown in Supporting Figure S1 for illustration.

### 3.1 Motion correction results

**Figure 2:**
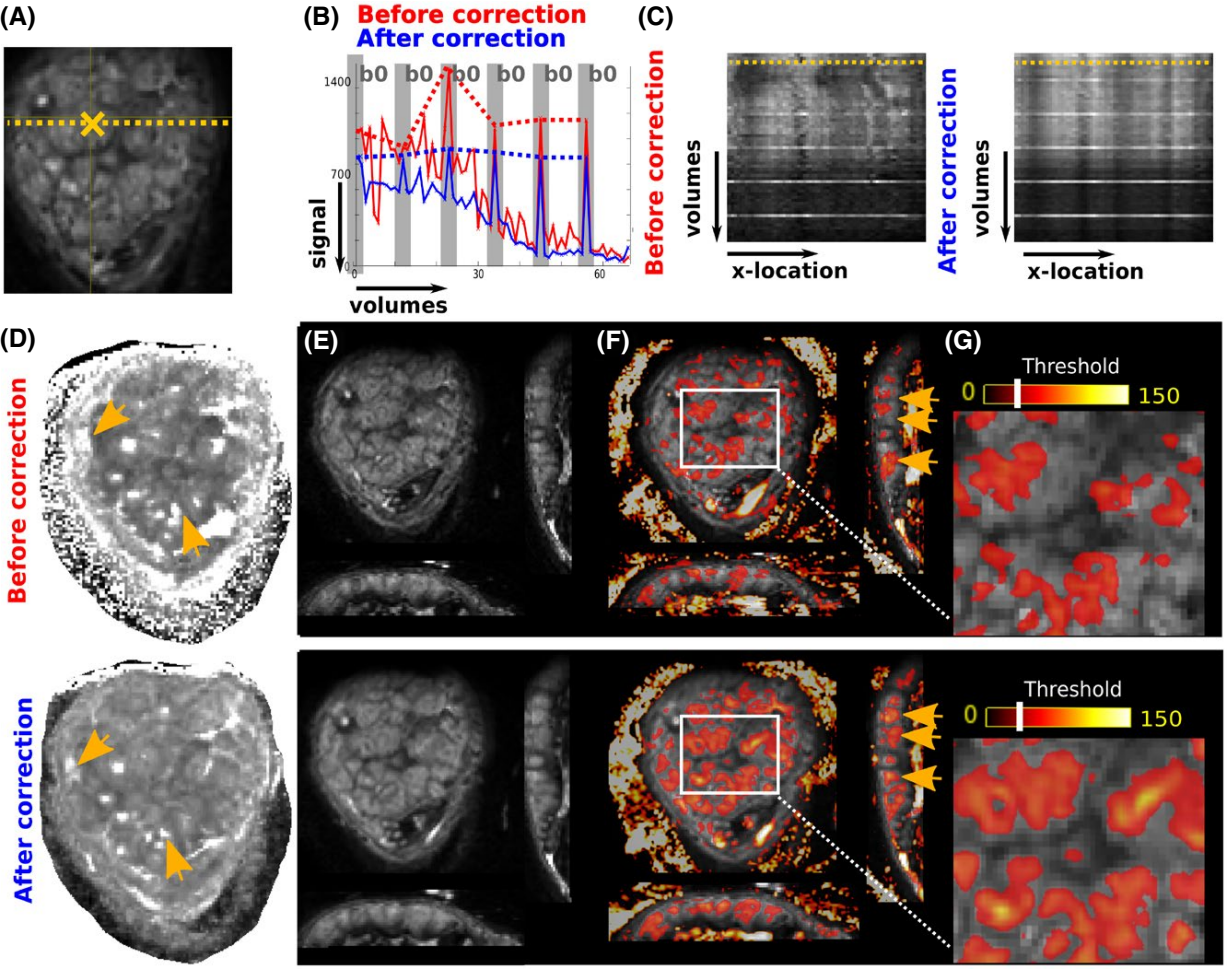
Results from the motion correction of the functional modalities are illustrated. (A) Coronal b0 slice from the diffusion scan to depict the locations analysed in (B) and (C): (B) The signal at the voxel marked by the orange cross in (A) is shown over all 64 volumes in (B). The relative differences in signal are due to the *b*‐value varying from time point to time point. The b0 volumes are indicated with vertical grey bars and the red and blue dotted lines track the signal for b0 values only. In (C) the signal intensity at the dotted line (A) is shown over volumes. In (D) the ADC map calculated before (top) and (after) motion correction is shown with the orange arrows indicating regions of improved localization and separation of high ADC areas. In (E) one b0 volume is shown before and after motion correction, (F) illustrates these b0 volumes with overlayed non‐corrected and corrected T2* maps (thresholded at 50). Both b0 and T2* maps are shown in all three planes (coronal, transverse, sagittal). The orange arrows indicate the better localization of high T2* regions within spatially separated higher signal areas in the spin‐echo signal. Finally in (G), a zoom into the central area indicated the increased spatial co‐localization of the high T2* areas with the anatomically visible lobules on the b0 images

Results from the motion correction of the functional modalities are illustrated in Figure 2(A) and (b). The signal at the voxel marked by the orange cross in (a) and selected as a lobule centre with high signal intensity in the uncorrected data is shown over all 64 volumes in (b) before and after motion correction. The b0 volumes are highlighted in grey and connected by a red (before correction) and blue (after correction) dotted line. The relative differences in signal are due to varying *b*‐value and thus persist after registration. However, within shells decreased signal fluctuations can be observed after motion correction illustrate that the chosen voxel depicts the same volume position over all volumes. The volumes acquired with *b* = 0 varied in signal strength before motion correction but achieve the same signal strength after motion correction as would be expected. The reduced signal amplitude after motion correction for the first volumes illustrates, that the chosen voxel—chosen as the lobule centre in the uncorrected data—has slightly shifted out of the centre. This behaviour is possible as not the first volume is chosen as registration target, but a template minimizing displacements over all volumes as described above.

In (c) the signal intensity at the dotted line in (a) over volumes similarly shows less fluctuation after motion correction. The ADC map (Figure 2(D)) calculated before (top) and after (bottom) motion correction depicts improved localization and separation of high ADC areas (orange arrows). In (e) one b0 volume is shown before (top) and after (bottom) motion correction in coronal and (reformatted) sagittal and transverse scanning plane. These same volumes are shown overlaid with non‐corrected and corrected T2* maps. Therefore, a threshold of 50 was applied to the T2* maps. The orange arrows in (f) indicate the better localization of high T2* regions within spatially separated higher signal areas in the spin‐echo signal. Finally, in (g) a zoom into a central placental regions is depicted before and after motion correction.

### 3.2 Structural results

**Figure 3:**
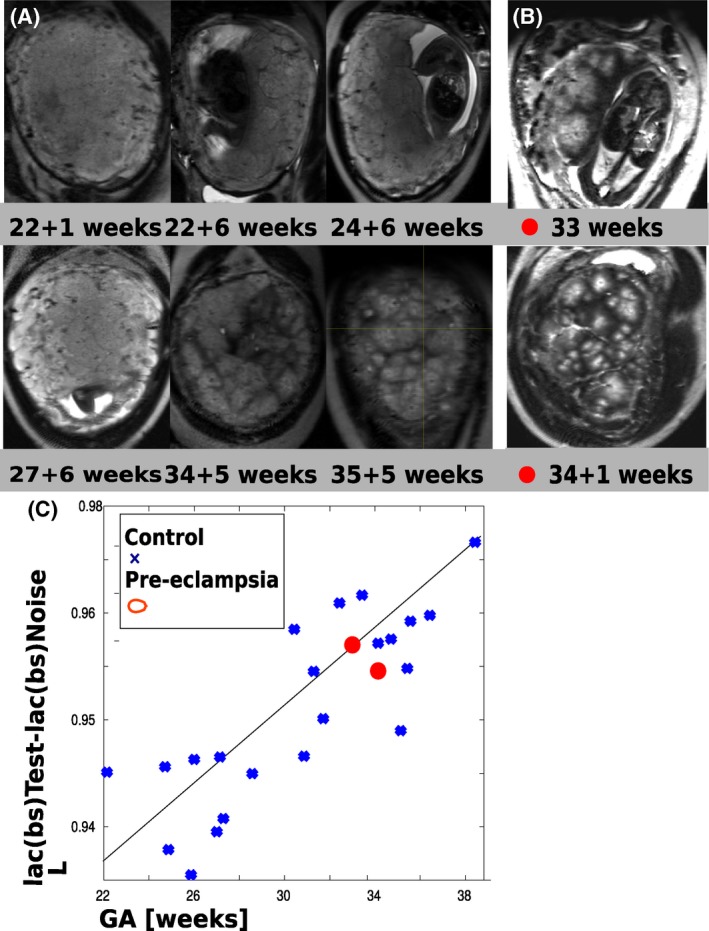
(A) Image results from the T2 weighted scans acquired on six volunteers from GA 22 + 1–35 + 3 weeks. For each, coronal planes at midpoint of the placental parenchyma between basal plate and chorionic surface are shown. Results from two (B) pre‐eclamptic placentas are depicted. (C) The results from the **L** measure are plotted vs. gestational age

Image results from the T2‐weighted scans are shown in Figure 3. Coronal slices from T2‐weighed acquisitions through the middle of the placental parenchyma (Figure 3(A)) for six control placentas with GA ranging from 22+1 to 35+3 weeks illustrate increased spatial heterogeneity and progressively clearer delineation of circular regions with decreasing signal from centre to periphery. While a certain spatial heterogeneity regarding the size of the high intensity regions can be observed, they are roughly of similar size across each individual normal placenta. Two placentas from PE cases are depicted in Figure 3(B), displaying hypointense region of diverse sizes but no clear common pattern.

For the lacunarity measure,^46^ results for preliminary tests conducted to determine the robustness and parameter settings are displayed in Supporting Information Figure S2(A) and (C) and discussed in the Supporting Information. Based on these experiments, the obtained lacunarity results are plotted against GA in Figure 3(C). The increase with GA corresponds well to the visual impression in Figure 3(A). The results from the PE‐placentas for this measure (marked by red dots) fall in among the values obtained from the control cases, indicating that the chosen measure is not sensitive to these clinical conditions.

### 3.3 T2* results

**Figure 4:**
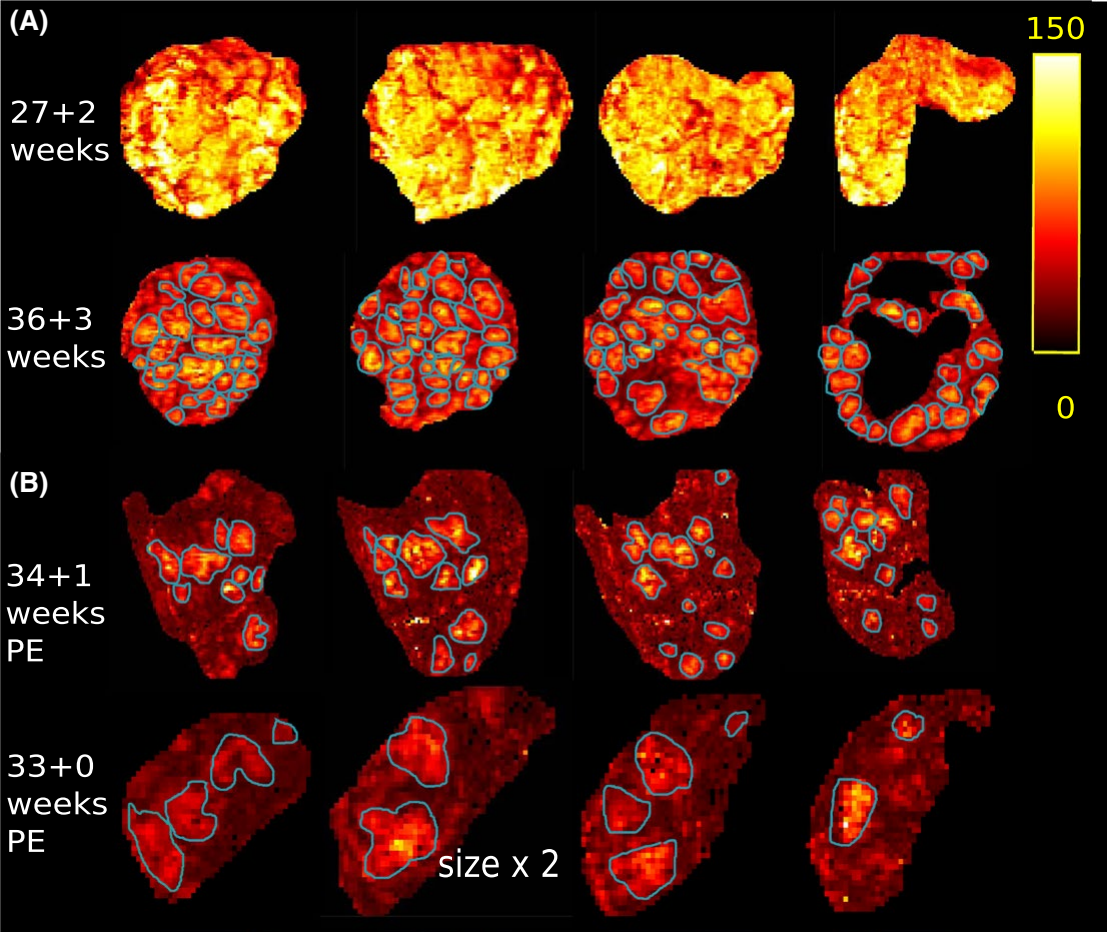
T2* results displayed in four coronal planes. (A) and (B) share the same T2* scale displayed on the right. (A) Images from two healthy volunteers at 27 + 2 and 36 + 3 weeks are shown. (B) Two T2* maps from pre‐eclamptic placentas at 34 + 1 and 33 + 0 weeks are depicted. Note that the second is depicted twice enlarged compared to the other placentas. The blue lines in (A) and (B) illustrate the perceived lobules delineation. The first row in (A) did not allow clear lobule delineation

Supporting Information S3 illustrates the first four echoes for three examples of the MEGE data. Nodular high T2* regions can be observed on the T2* maps (Figure 4(A)) surrounded by regions of spatially rapidly decaying signal. While some variability in size can be observed between these regions, they display roughly similar sizes and T2* values in their centres. Over gestation, an increase in T2* ratio between centres and surrounding hypo intense areas becomes evident, leading to a perception of an apparently smaller diameter. The PE placentas in Figure 4(B), illustrate isolated high T2* regions with rapidly decaying periphery. Individual high T2* regions are segmented manually and delineated by blue lines in (a) and (b). Thereby, the first row in (a) did not allow this, as no clear lobule boundaries can be seen yet. As can be seen in this example, the PE placentas display areas of substantially reduced T2* and do not have the typical lobular appearance.

**Figure 5:**
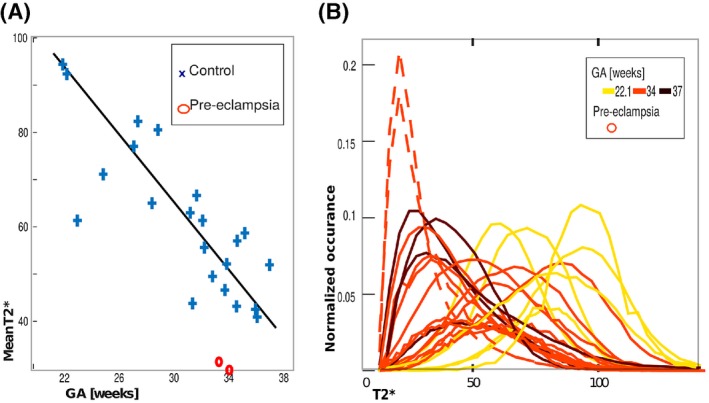
(A) The mean T2* over gestation is illustrated in blue for healthy and red for the pre‐eclamptic placentas. (B) The histogram distributions normalized by placental volume of healthy and pre‐eclamptic placentas are depicted with colour‐coded gestational age (bright yellow: 20 weeks to dark red: 40 weeks). The dotted line indicates PE cases

Quantitative whole‐organ measures from the T2* maps are shown in Figure 5(A) and (B). The whole‐organ mean T2* decreases over gestation (90 ms at 20 weeks to 25 ms at 40 weeks) as shown in Figure 5(A). These are indicative of a change from a homogeneous T2* distribution resulting from slow T2* decay from centre to periphery to a more heterogeneous T2* appearance with small central high T2* regions. The two PE placentas have a decreased mean T2*.

Histograms normalized for placental volume (Figure 5(B)) provide more detailed evaluation but also illustrate the high inter‐placental variability. In this figure the colour corresponds to GA (bright yellow = 20 weeks to dark red = 40 weeks). The volume‐normalized histograms display a general shift to the left (decreasing mean T2*) with increasing GA, and this is reflected in Figure 5(B) where there is a fall from around 80 ms at 20 weeks to 30 ms at 40 weeks. These are suggestive of a change from a heterogeneous T2* distribution (larger width) resulting from slow T2* decay from centre to periphery with the regions of high T2* covering the entire parenchyma to a more uniform low‐T2* appearance with spatially small areas with high T2* in the centres. Both PE placentas can be identified by a more pronounced and left‐shifted peak.

The reproducibility of the obtained T2* maps was investigated with 12 additional scans, where the MEGE sequence was repeated either in the same session or after repositioning and a novel shimming in the second session. The results are illustrates in Figure S4.

### 3.4 Microstructure results

**Figure 6:**
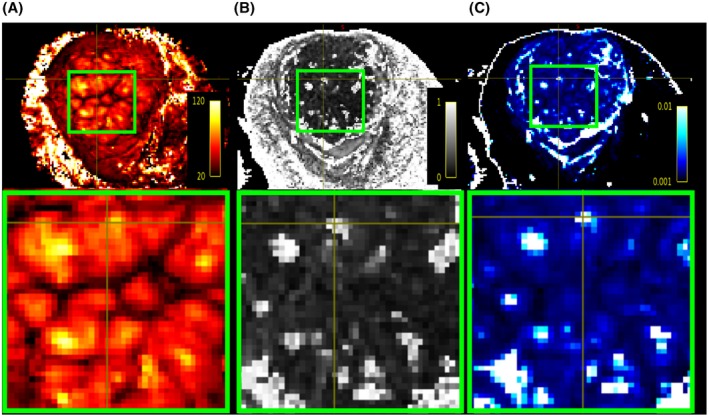
Result of the registered MEGE and dMRI results. The same mid‐placental slice is shown for the (A) T2* map, the (B) fractional anisotropy and (C) apparent diffusion coefficient. A zoom into a central region with 9 lobules is shown in the lower row

Figure 6 shows a zoom into a central placenta region depicted on the T2^*^ map alongside the FA map (Figure 6(B)) and ADC map (Figure 6(C)), all illustrating an inhomogeneous pattern. Many regional centres exhibit spatially co‐located patches of high ADC and high FA (Figure 6(B) and (C)). Since we fit the diffusion tensor to all *b*‐values, these maps combine perfusion and diffusion effects. Therefore these patches of increased ADC and FA may reflect the central cavity in which inflow of maternal blood with coherent orientation occurs. This conclusion is supported by the corresponding high T2* in Figure 6(A). Areas of high FA are also consistent with highly directional tissue microstructure due to the affects of this inflow on the growing fetal villous tree.

**Figure 7:**
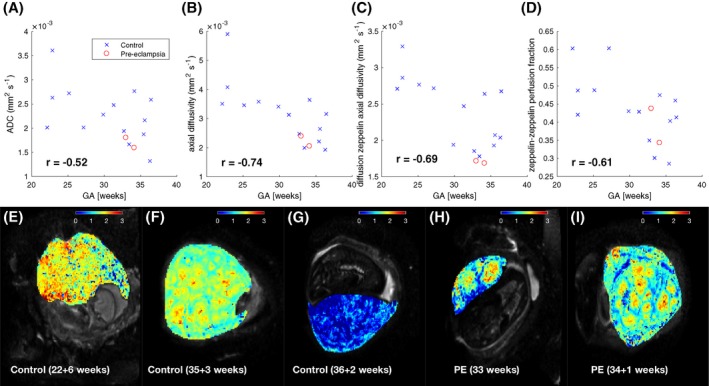
Mean of dMRI‐derived parameters in placenta ROI across gestation: (A–B) show parameters derived from a diffusion tensor model fit, whereas (C–D) show zeppelin‐zeppelin parameters. (E–I) Maps of diffusion zeppelin axial diffusivity (units × 10^−^3 mm^2^ s^−^1), derived from zeppelin‐zeppelin model fits for control (E–G) and pre‐eclampsia (H–I) cases

Diffusivity measures in the placenta ROI, and zeppelin‐zeppelin perfusion fraction in the basal plate ROI all decrease across gestation (Figure 7(A)–(D)), suggesting that they are sensitive to changes in microstructure and microcirculation during to placental maturation. The axial diffusivities show the strongest trend, with ADC much weaker although we emphasize the small number of samples.

These organ‐averaged statistics do not discriminate pre‐eclampsia from controls, unlike T2*, but maps of the diffusion zeppelin (i.e. *D*) axial diffusivity (Figure 7(E)–(I)) show a very heterogeneous pattern for PE cases, with large areas of very low diffusivity.

A previous bootstrapping analysis for an anisotropic IVIM model similar to zeppelin‐zeppelin showed that estimated parameter standard deviations were typically 10 times smaller than parameter values,^48^ suggesting that we can indeed confidently infer regional contrast.

## 4 DISCUSSION AND CONCLUSIONS

This paper addresses the challenge to achieve further steps towards a non‐invasive assessment of the human placenta combining several sub‐functions in a clinically feasible scan time. Therefore, the study was designed to include three key techniques to visualize macrostructure, microstructure and oxygenation across the whole placenta and along gestation to allow qualitative and quantitative exploration of placental physiology, disease phenotype and aetiology. A multi‐modal functional placental MRI acquisition composed of anatomical imaging, diffusion imaging and T2*‐relaxometry in 15 min was presented. All modalities are set‐up to allow whole organ assessment over the full gestational period, and have been demonstrated from 20 weeks to term age. Preliminary experiments were conducted to tune key parameters to be sensitive to the expected function. This includes the echo times for T2* imaging and the *b*‐values and vectors for diffusion encoding. The acquired data is most powerfully exploited with dedicated post‐processing and quantification steps. A possible processing pipeline was presented in this study.

The obtained results from all three studied modalities illustrate in different ways the importance of the placental subunits: Only accompanying histo‐pathological data could confirm that the low signal areas correspond indeed to septa delineating anatomical units. However the key features of the units, including number (10–30), size (1–4 cm), evolution over gestation, clearer delineation (Figure 3) and T2* decay from centre to periphery (Figure 4), alignment (Figure 6), and spatial parameter maps (Figure 7) point towards this conclusion and can provide discriminating insights.

The quantitative lacunarity results in Figure 3(C) correlate well to the visual impression of an increase in both granularity and lobularity. The proposed lacunarity measure is designed to pick up intensity features 2 cm × 2 cm in area, which corresponds to the expected size of the anatomical units between the septa. Only the combination with pathological analysis could confirm beyond doubt that the increasing ratio between centre and surrounding tissue depicts increasing differentiation between oxygen‐rich lobules and more and more fibrin‐deposition in the septa. The demonstrated correlation with gestational age suggests however, that this measure is sensitive to maturation of the placenta.

While physiological and pathological placental maturation assessments based on the sonographic classification of Grannum^49^ have been used previously, they have demonstrated poor inter‐observer agreement and poor predictive value for perinatal outcome.^15^ An alternative (automated) MRI based measure in combination with other functional features might thus be of substantial interest. This goes beyond previous MRI‐based studies assessing placental maturation, which were limited to qualitative assessment.^50^, ^51^

There are, however, several limitations in the preliminary texture analysis presented. The lacunarity measure merely corresponds to a first attempt to use quantitative texture features on anatomical placental images. Its robustness with regard to variations in data acquisition parameters such as choice of echo time or chosen imaging sequence was not assessed in this study, and only T2 weighted images, obtained with 2D Turbo Spin Echo with an echo time of 180ms on a 3T scanner were tested. Influence of box size and robustness to slice selection were investigated in preliminary exploratory experiments. These constitute, however, no thorough evaluation of the entire parameter space. No further texture features have been tested and compared.

In addition, a potential limitation of the presented measure is the choice of a mid‐parenchymal slice. This was chosen as a simple means to address the maturation within the very core of placental function—the inter‐villous space—and its stability was assessed in Supporting Information Figure S2A. This could, however, be potentially improved by including further slices.

The sharp decline in T2* from centre to periphery in every lobule corresponds well with the physiological transfer of oxygen from the maternal blood to the fetal circulation within the intervillous space. High T2* regions with two apparent centres might correspond to anatomical units with two spiral artery inlets—each surrounded by areas of high T2*. The focal higher T2* in smaller lobules through gestation would then be a consequence of the denser villous trees and therefore optimized quicker uptake of oxygen from maternal to fetal blood and thus spatially closer increase of deoxyhemoglobin around the maternal inlets. The importance of the slope of this decay was shown in a rhesus monkey model,^33^ and their published results correspond visually well to our data. This supported the notion that this decay slope depends on ‐and thus reveals‐ maternal supply and fetal intake parameters (blood flow velocity, permeability, surface area), which inform on anatomical features (spiral artery remodelling, fetal vasculature).^39^ Spatial interpretation of these results on an individual lobule level, such as performed by^33^ will be a next step. The shown decreasing T2* over gestation correlates well with previous results.^19^

It seems that the T2* measures, and specifically those identifying the fraction of “active” (oxygen‐rich) voxels within the placenta could enable visualization of specific features of pre‐eclampsia. All results regarding the pre‐eclamptic placentas, are very preliminary due to the small sample size of 2. The observed decrease and left shift in the histogram is, however, in line with previous results from placentas complicated by fetal growth restriction.^20^, ^22^

The advanced placental diffusion MRI acquisition allowed us the calculation of standard diffusion measures as in previous studies,^30^ but offers at the same time data with high angular resolution, a wide spread in diffusion strength and high spatial resolution, well suited for more advanced micro‐structural modelling approaches.^41^ The presented measures showed trends over gestation and seemed not to be sensitive to placental changes in pre‐eclampsia. Unlike T2*, tensor‐derived placental diffusivity metrics do not follow a clear trajectory over gestational age.

This suggests that either: dMRI‐derived measures alone are not well‐suited for placental characterization, or that the models we utilized are not sensitive enough to adequately capture pathological or developmental changes. This highlights a potential need to utilize more specific dMRI‐derived measures of placental microstructure, perhaps in combination with information from other MRI modalities, to increase overall sensitivity to placental viability. Furthermore, the demonstrated increase in heterogeneity in both pre‐eclamptic placentas illustrates the importance of the whole‐organ approach.

In this study, we utilized a dMRI model (i.e. zeppelin‐zeppelin) which was previously developed specifically for the placenta.^48^ However, it is still unclear exactly how the unique anatomy of the placenta affects the diffusion MRI signal, and hence what type of dMRI models have most utility. The extent to which the dMRI models utilized here realistically represent this unique anatomy is an open question. A major unresolved issue is whether maternal blood within intervillous space has the most effect on the perfusion or diffusion compartments , or even if it is reasonable to think in terms of separate compartments (see^48^ for a fuller discussion). There is therefore huge scope for further development of placental dMRI models.

The proposed registration technique accounts for the non‐rigid placental motion as a result of maternal breathing and fetal motion and allows the different modalities to be placed in a common reference space (Figures 2 and 6). Previous placental registration approaches have successfully registered intra‐modality volumes^32^, ^52^ but to our knowledge not attempted inter‐modality registrations. Combination with super‐resolution slice‐to‐volume reconstruction techniques^37^ has the potential to be beneficial and the needs of these approaches is respected in our acquisition. It furthermore supports slice‐level motion correction techniques as proposed recently.^53^

General limitations of this study are the exclusion of posterior placentas from the quantification and the chosen cross‐sectional approach. The reason to include only placentas with anterior and fundal location was the consistently worse image quality on posterior placentas (See Supporting Information Figure S1). This can be explained by the less favourable position of posterior placentas within the maternal abdomen in relation to the MRI surface array coils used. The inclusion of all placentas independent of location is a necessary next step and could potentially be enabled by either use of more customized surface coils^54^ or further pre‐processing including filtering to allow quantification results to be independent of placental location.

Another limitation of this study is the choice of the cross‐sectional vs longitudinal approach. This decision was driven by the limited availability of longitudinal data on pregnant women and difficulties in recruiting participants with pregnancy complications early enough to allow two separate serial MRI scans. Longitudinal data would enable an even more specific study of for example, compensation mechanisms and disease aetiology. The already observed trends over gestation (increased lacunarity, decreased mean T2* and decreased axial diffusivity) provide evidence to support the design of more demanding longitudinal approaches in the future. The developed acquisition protocol, post‐processing and quantification can and will be employed in any such future longitudinal opportunities.

To summarize, a multi‐modal functional MRI approach is presented, which offers assessment of the human placenta in‐vivo throughout gestation. The individual modalities, the developed processing pipeline and data across gestation are shown as well as its ability to inform on normal and pathological placental development on several levels. The obtained data suggests that the lobule delineation, perfusion, oxygen exchange as well as microstructure can be visualized.

Exemplary data sets including all three modalities as well as script for our full processing pipeline will be available on our institution's website to facilitate the development of further post‐processing and quantification.

## Supporting information


**FIGURE S1**Unprocessed b0‐diffusion MRI data from a lateral placenta which was excluded from the quantification. The coronal view illustrates an anterior and posterior slice both with the same scaling.
**FIGURE S2** (a) The lacunarity measures for different boxsizes are shown for both a placenta ROI with placental tissue properties (red line) and with random noise (blue line). (b) Results from the chosen L measure is shown for several slices over an exemplary placenta. (c) The L results are shown for different volumes, illustrating a stable, volume‐independent value for objects above 10000 voxels.
**FIGURE S3** Image results from the Multi‐echo Gradient Echo scans acquired on three volunteers at GA 22+1 weeks (first row), 35+3 weeks (second row) and 30+3 weeks (third row). For each, three planes are shown, the native coronal plane, and the reformatted axial and sagittal planes. The coronal plane was chosen approximately halfway between the basal and chorionic plate. The first four TEs are shown in (a)–(d), the obtained proton density map in (e) and the T2* map in (f).
**FIGURE S4** Quantitative results from a repeatability test for the T2* maps. The MEGE sequence was either repeated in the same session (1 or 2) or for five of the 12 illustrated datasets in separate sessions. Between sessions, the patient leaves the scanner and a new image‐based shim is calculated. T2* maps were calculated, masked and the mean value displayed. In (b) the obtained slices for one example in both sessions are depicted.
