# Supplementary material for: Multi‐modal functional MRI to explore placental function over gestation
Source: Magn Reson Med. 2018 Sep 21;81(2):1191–204. doi: 10.1002/mrm.27447 (PMC6585747; doi:10.1002/mrm.27447)
Supplement: Supplementary file 1 — FIGURE S1Unprocessed b0‐diffusion MRI data from a lateral placenta which was excluded from the quantification. The coronal view illustrates an anterior and posterior slice both with the same scaling. FIGURE S2 (a) The lacunarity measures for different boxsizes are shown for both a placenta ROI with placental tissue properties (red line) and with random noise (blue line). (b) Results from the chosen L measure is shown for several slices over an exemplary placenta. (c) The L results are shown for different volumes, illustrating a stable, volume‐independent value for objects above 10000 voxels. FIGURE S3 Image results from the Multi‐echo Gradient Echo scans acquired on three volunteers at GA 22+1 weeks (first row), 35+3 weeks (second row) and 30+3 weeks (third row). For each, three planes are shown, the native coronal plane, and the reformatted axial and sagittal planes. The coronal plane was chosen approximately halfway between the basal and chorionic plate. The first four TEs are shown in (a)–(d), the obtained proton density map in (e) and the T2* map in (f). FIGURE S4 Quantitative results from a repeatability test for the T2* maps. The MEGE sequence was either repeated in the same session (1 or 2) or for five of the 12 illustrated datasets in separate sessions. Between sessions, the patient leaves the scanner and a new image‐based shim is calculated. T2* maps were calculated, masked and the mean value displayed. In (b) the obtained slices for one example in both sessions are depicted. [file MRM-81-1191-s001.pdf]

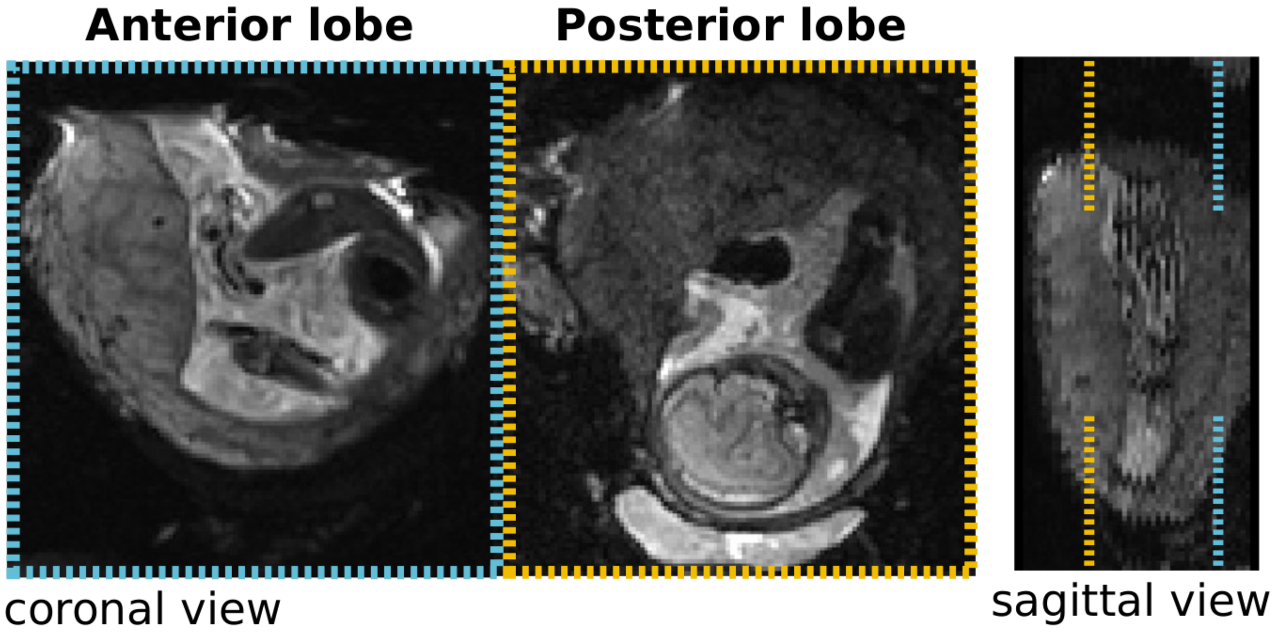

Supporting Information Figure S1: Unprocessed b0-diffusion MRI data from a lateral placenta which was excluded from the quantification. The coronal view illustrates an anterior and posterior slice both with the same scaling.

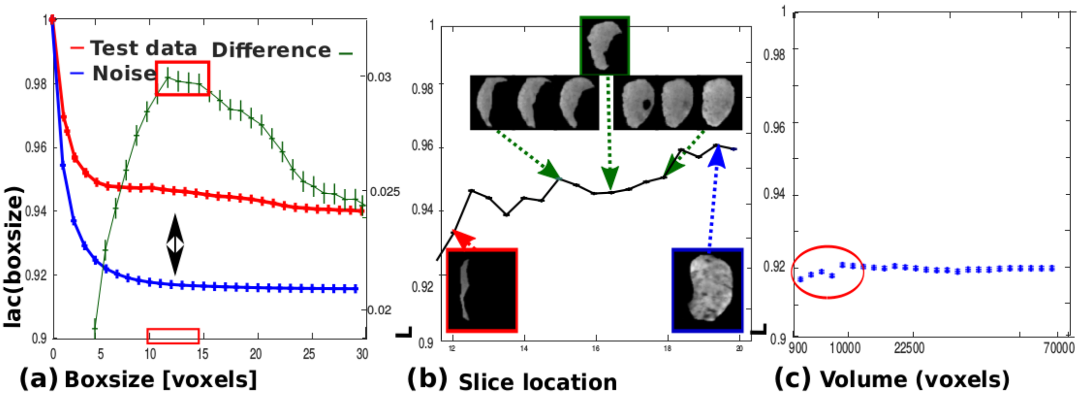

Supporting Information Figure S2: (a) The lacunarity measures for different boxsizes are shown for both a placenta ROI with placental tissue properties (red line) and with random noise (blue line). (b) Results from the chosen L measure is shown for several slices over an exemplary placenta. (c) The L results are shown for different volumes, illustrating a stable, volume-independent value for objects above 10000 voxels.

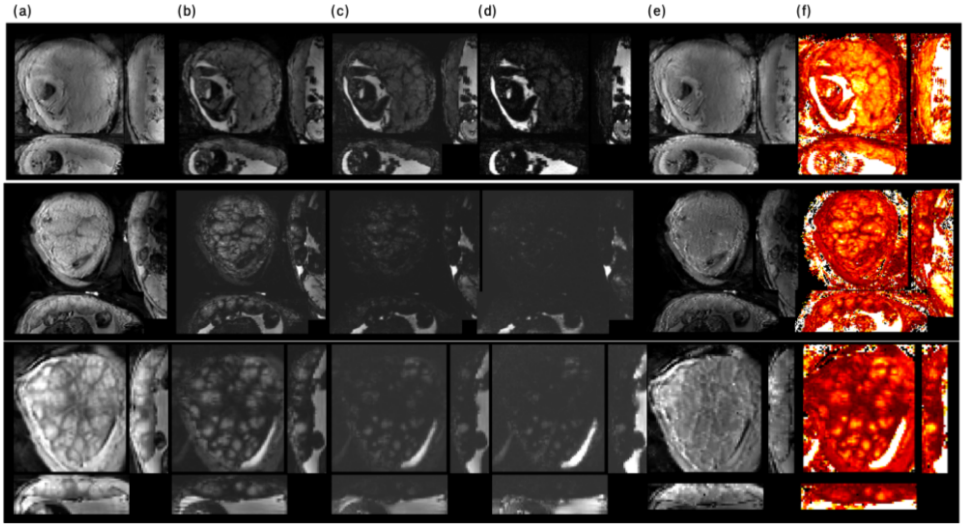

Supporting Information Figure S3: Image results from the Multi-echo Gradient Echo scans acquired on three volunteers at GA 22+1 weeks (first row), 35+3 weeks (second row) and 30+3 weeks (third row). For each, three planes are shown, the native coronal plane, and the reformatted axial and sagittal planes. The coronal plane was chosen approximately halfway between the basal and chorionic plate. The first four TEs are shown in (a)-(d), the obtained proton density map in (e) and the T2* map in (f).

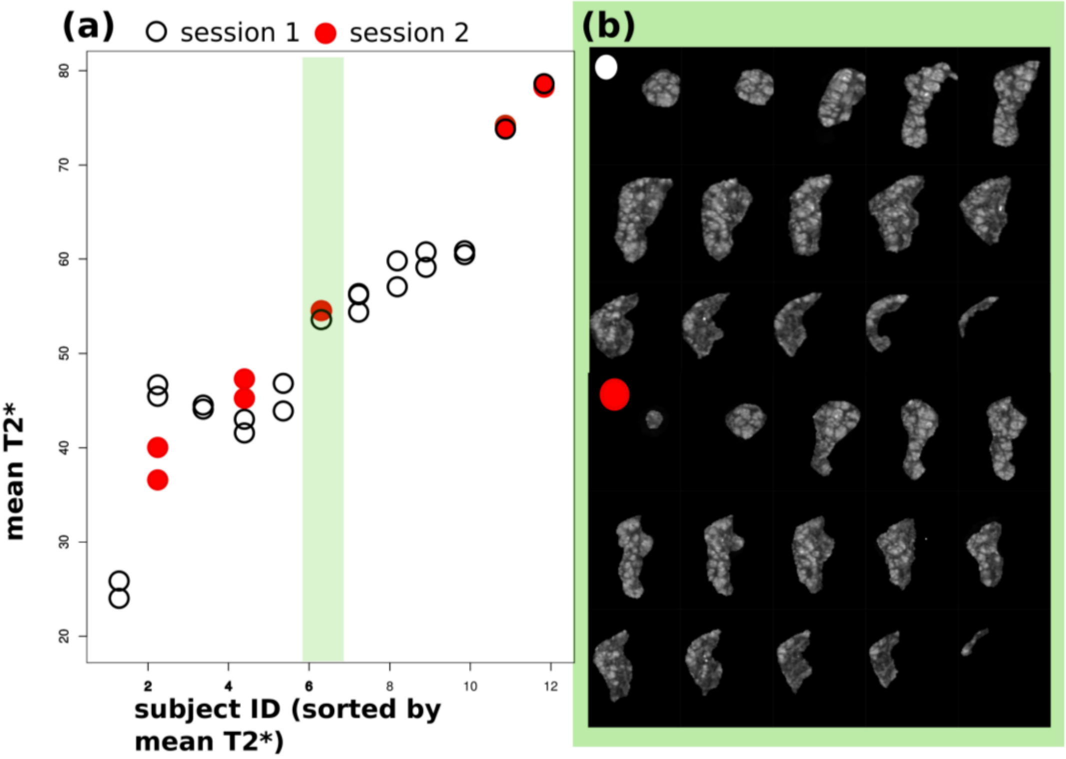

Supporting Information Figure S4: Quantitative results from a repeatability test for the T2* maps. The MEGE sequence was either repeated in the same session (1 or 2) or for five of the 12 illustrated datasets in separate sessions. Between sessions, the patient leaves the scanner and a new image-based shim is calculated. T2* maps were calculated, masked and the mean value displayed. In (b) the obtained slices for one example in both sessions are depicted.

Lacunarity method The steps performed to obtain the lacunarity results shown above are detailed in the following:
Here, the lacunarity (lac(b)) was calculated with box sizes in steps of $[2-30]^2$ voxels ($[3-30]^2$ mm^2) as follows:
For a boxsize b, the mass M is calculated for every possible of the $N(b)$ boxes with size b as the total number of voxels above the mean in the box. Thereby, only boxes lying completely within the segmented area are taken into account. In the next step, the mass distribution $n(M,b)$ is calculated over the entire image as the number of boxes of size b with mass M ($M = 0,..,b^2$) and the probabilitity distribution $Q(M,b)$ is obtained as $Q(M,b) = n(M,b)/N(b)$.
Finally, the lacunarity $lac(b)$ is defined as

$$lac(b) = \sum M^2 Q(M,b)/(\sum MQ(M,b))^2. \tag{1}$$

First, the ideal boxsize to detect the expected texture was explored. To do this, an exemplary placenta slice with the true texture and the placental volume filled with texture-free random noise were analysed with boxsizes (b) between $b = 2$ and $b = 30$ voxels (red and blue line in (a)). The results and especially the difference (green line) in (a) illustrate, that a boxsize of $11^2 - 14^2$ provide the strongest differentiation between texture and noise. Since the noise level is highly variable between different placenta examinations, it is helpful to normalise by $lac(2)$, which captures short range fluctuations that reflect noise levels. Therefore, in the following, the measure **L** is calculated as

$$\mathbf{L} = \sum_{b=11}^{b=14} lac(s)/lac(2). \tag{2}$$

To determine the influence of the slice location, **L** was calculated for each image slice from a healthy normal placenta after segmentation to exclude non-placenta tissue from the calculation. The results in (b) illustrate, that a a stable result was achieved within all 7 central slices around the chosen mid-parenchymal slice (green arrows) with $\mathbf{L} = 0.948 \pm 0.0024$. The results, are, however, more variable at the borders of the placenta, probably due to the decreased size (red arrows) and the inclusion of the vessels in the basal plate (blue arrow). Next, and following from this, to ensure, that the texture is analysed, rather then the size of the considered tissue within the acquired slice, the size of the region of interest was varied between 30^2 to 270^2 voxels. The results in (c) illustrate stable **L** results for volume sizes above 10000 voxels.
